# Sugar-Specific Modulation of Trophoblast Function and Bioenergetics Under Metabolic Stress Conditions

**DOI:** 10.3390/ijms27198920

**Published:** 2026-10-08

**Authors:** Elizabeth Thurmond, Madeline Boyer, Benjamin T. Bikman, Paul R. Reynolds, Juan A. Arroyo

**Affiliations:** 1 Department of Cell Biology and Physiology, Brigham Young University, Provo, UT 84602, USA; 2 Department of Preparatory Medicine, Brigham Young University School of Medicine, Provo, UT 84602, USA

**Keywords:** trophoblast function, placental metabolism, oxidative stress, sugar alternatives, gestational diabetes

## Abstract

How sugars other than glucose affect trophoblast function is poorly understood, particularly under the metabolic stress of diabetic pregnancy. We compared glucose, fructose, and the minimally metabolized sugar allulose in two trophoblast cell lines, SW71 (first-trimester, extravillous-like) and BeWo (choriocarcinoma-derived, villous). Each sugar was added at 25 mM for 24 h to the cells’ standard glucose-containing media (11 mM, SW71; 17.5 mM, BeWo) to model metabolic stress. All three sugars increased the number of cells recovered (2.4- to 6.5-fold in SW71; ~10- to 15-fold in BeWo) and reduced viability (~9–75%), with fructose causing the largest loss in both lines. Intracellular reactive oxygen species increased 5.2- to 7.8-fold with all sugars. Oxygen consumption differed by sugar: in SW71 cells, glucose and allulose increased ADP-stimulated and succinate-supported respiration, whereas fructose reduced it, and in BeWo cells only fructose increased it. SW71 invasion was ~7-fold higher after allulose than after glucose or fructose. BeWo cells were studied undifferentiated, so effects on syncytialization were not assessed. The three sugars are therefore not interchangeable in their effects on trophoblast cells in vitro. Because the concentrations exceed those in maternal blood, these findings are hypothesis-generating and require testing at physiological concentrations and in primary trophoblast models.

## 1. Introduction

Pregnancy is characterized by profound physiological adaptations that support fetal growth and development, many of which are coordinated by the placenta. This transient organ functions as the primary interface between maternal and fetal compartments, regulating nutrient and gas exchange, hormone production, immune tolerance, and vascular remodeling. The successful establishment and maintenance of placental function depend on the differentiation and activity of trophoblast cells, which arise from the trophectoderm of the blastocyst and form the outermost layer of the placenta throughout gestation [1]. Because placental development must proceed in parallel with rapidly changing maternal physiology, trophoblast cells are particularly sensitive to alterations in the metabolic environment.

Trophoblast cells differentiate into distinct lineages with specialized roles. Villous cytotrophoblasts (CTBs) proliferate and fuse to form the multinucleated syncytiotrophoblast (STB), which covers the placental villi and directly contacts maternal blood. The STB is responsible for nutrient transport, endocrine activity, and barrier functions essential for fetal survival [2]. In parallel, a subset of CTBs differentiates into extravillous trophoblasts (EVTs), which invade the decidua and remodel uterine spiral arteries to ensure adequate uteroplacental blood flow [3]. These divergent trophoblast lineages therefore operate under markedly different energetic and signaling demands. Disruption of trophoblast differentiation or function is associated with pregnancy complications including preeclampsia, fetal growth restriction, and gestational diabetes mellitus (GDM) [4].

Among the nutrients transported across the placenta, carbohydrates—particularly glucose—play a central role in fetal development. Glucose is the primary energy substrate for the fetus, which has limited capacity for endogenous glucose production and is therefore highly dependent on maternal supply [5]. Placental glucose transfer occurs predominantly via facilitated diffusion mediated by members of the solute carrier family 2 (SLC2), commonly referred to as glucose transporters (GLUTs) [6]. Of these, GLUT1 is the most abundant isoform in the human placenta and is expressed on both the microvillous (maternal-facing) and basal (fetal-facing) membranes of the syncytiotrophoblast [6]. However, transporter expression alone does not fully explain variation in placental glucose handling across gestation or disease states [6].

The asymmetric distribution and regulation of GLUT1 across the syncytiotrophoblast membranes are critical determinants of transplacental glucose flux [7]. Additional GLUT isoforms, including GLUT3 and GLUT9, have been identified in trophoblast cells and may contribute to glucose uptake and intracellular metabolism, particularly during early gestation or under conditions of altered nutrient availability [8]. Placental glucose transport capacity increases as pregnancy progresses, reflecting both structural expansion of the villous surface area and gestational regulation of transporter expression [9].

Trophoblasts exhibit high metabolic activity, relying on glucose via glycolysis, oxidative phosphorylation, and the pentose phosphate pathway to support Adenosine Tri-phosphate (ATP) production, biosynthetic pathways, hormone synthesis, redox balance, and nucleotide synthesis necessary for proliferation and differentiation [10,11]. The relative contribution of these pathways varies with trophoblast subtype and differentiation status, suggesting that metabolic regulation is closely coupled to trophoblast function rather than reflecting uniform energy demand. Emerging evidence indicates that sugar availability influences trophoblast behavior beyond energy metabolism. Exposure of trophoblast cells to altered glucose concentrations has been shown to modulate trophoblast proliferation, differentiation, and endocrine signaling [12]. In particular, high glucose conditions, relevant to diabetic pregnancies, affect trophoblast gene expression, oxidative stress responses, and inflammatory signaling pathways [13]. Moreover, glucose levels can influence the release and cargo of trophoblast-derived extracellular vesicles, which play a role in maternal–fetal communication and vascular adaptation during pregnancy [8].

The regulation of placental glucose handling is therefore closely intertwined with maternal metabolic status. In pregnancies complicated by diabetes or obesity, placental glucose transport is often dysregulated, contributing to excessive fetal glucose exposure and altered fetal growth trajectories [14]. These observations support the concept that altered placental nutrient handling contributes to developmental programming, although the specific cellular pathways involved remain incompletely defined [15].

Despite extensive study of placental glucose transport, far less is known about how trophoblast cells respond to different extracellular sugars or whether such responses vary across trophoblast lineages. Most in vitro studies implicitly treat sugars as metabolically equivalent substrates, despite clear differences in uptake pathways and downstream metabolic fates. Moreover, comparative analyses examining extravillous and villous trophoblast responses under identical sugar conditions are limited. Addressing these gaps is essential for understanding how changes in maternal carbohydrate availability may differentially affect trophoblast function and placental development, particularly in both normal and metabolically complicated pregnancies. Although the sugar concentrations used in experimental models often exceed physiological maternal glucose levels, such exposures are commonly employed to model metabolic stress and to reveal pathway-specific cellular responses.

Among dietary sugars, allulose (D-allulose, also termed D-psicose) has attracted growing interest as a comparator of metabolic relevance. A rare monosaccharide and the C-3 epimer of fructose, allulose occurs naturally in small quantities in foods such as figs, raisins, and wheat, and is produced commercially as a low-calorie sweetener with approximately 70% of the sweetness of sucrose but negligible caloric and glycemic impact [16,17]. Despite its increasing incorporation into the food supply, allulose currently lacks pregnancy-specific dietary or safety guidance: no human data on allulose exposure during pregnancy have been published, and regulatory evaluations (e.g., its Generally Recognized as Safe status in the United States) have not established pregnancy-specific intake recommendations, with the available reproductive and developmental data limited to animal studies reporting no teratogenic effects at high oral doses [18]. Its negligible caloric impact reflects its biochemistry: unlike glucose or fructose, allulose is minimally phosphorylated by hexokinase or fructokinase, does not appreciably enter glycolysis, and is largely absorbed and excreted unchanged in the urine, such that any cellular effects are unlikely to arise from its use as a glycolytic substrate. Because allulose is marketed to people managing body weight or glycemia, maternal exposure during pregnancy is plausible, yet its effects on trophoblast biology have not been studied. We therefore included allulose as a comparator alongside glucose and fructose to test whether a minimally metabolized monosaccharide elicits trophoblast responses distinct from those of the two principal dietary sugars.

We hypothesized that exposure to different extracellular sugars would differentially affect trophoblast cell number and viability, oxidative stress, mitochondrial respiration, and invasive capacity, and that these effects would differ between two trophoblast models. To test this, we examined the effects of glucose, fructose, and the rare sugar allulose on Swan71 (SW71) and BeWo trophoblast cells under conditions of metabolic stress.

## 2. Results

### 2.1. Glucose, Allulose, and Fructose Increase Trophoblast Cell Numbers but Impair Viability

**Figure 1:**
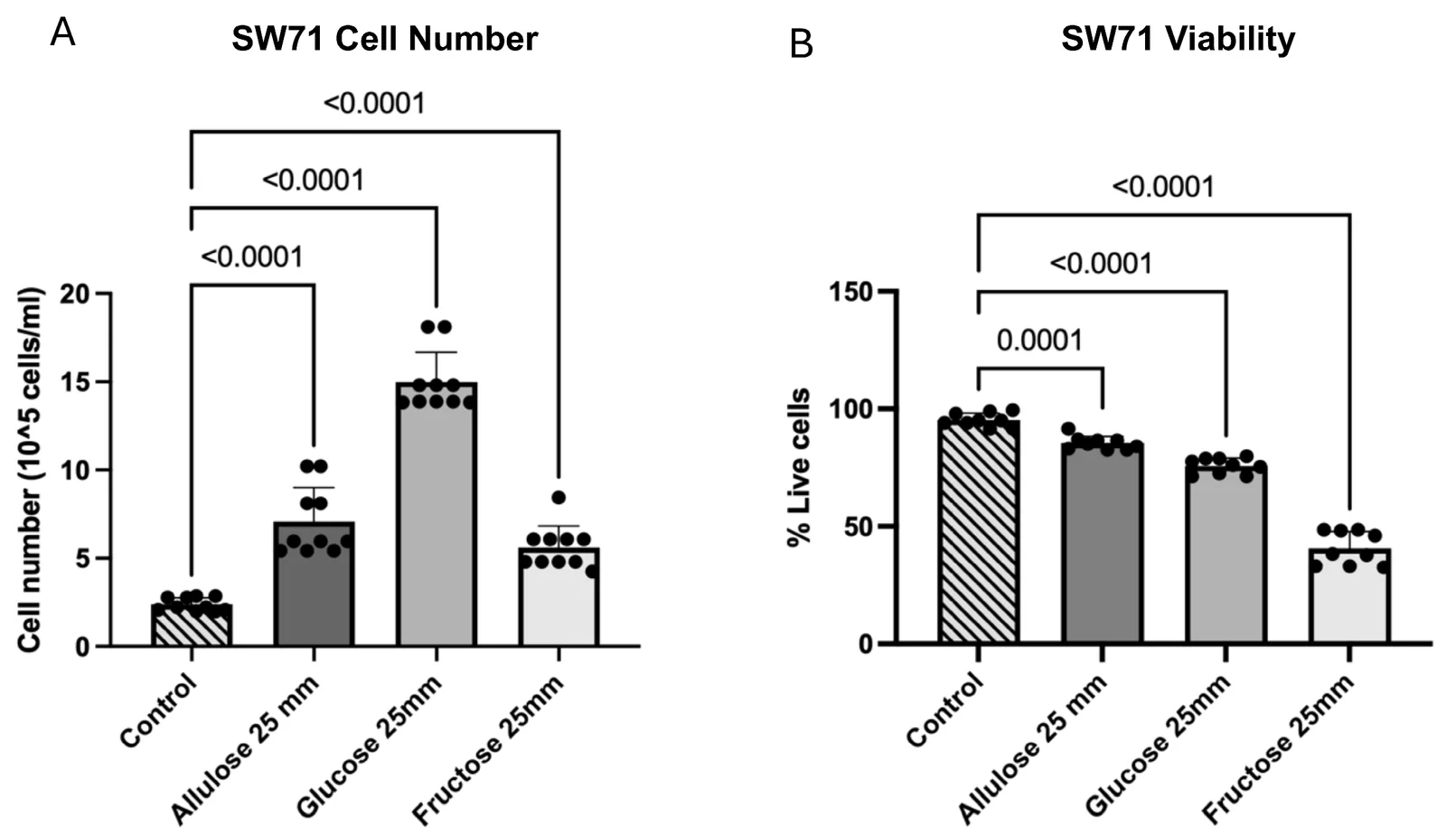
Effects of glucose, allulose, and fructose on SW71 trophoblast cell number and viability. SW71 extravillous trophoblast-like cells were treated for 24 h with glucose, allulose, or fructose added at 25 mM to standard medium (RPMI, 11 mM basal glucose); controls received standard medium alone. (**A**) Total cell number was significantly increased by all sugar treatments compared with the control. (**B**) Cell viability, determined by Trypan Blue exclusion, was significantly reduced in all sugar-treated groups. Individual data points are shown as black dots. Data are mean ± SEM (n = 10 independent experiments). Statistical analysis was performed using the Kruskal–Wallis test with Dunn’s post hoc correction.

We first looked at how *Glucose, Allulose and Fructose* affect cell numbers and survival in our trophoblast models. Fold-changes in cell number are expressed relative to the untreated control at 24 h. In invasive SW71 cells, all three sugars significantly increased cell counts compared with controls, with the largest increase following glucose (6.5-fold; *p* < 0.0001), followed by allulose (3.2-fold; *p* < 0.0001) and fructose (2.4-fold; *p* < 0.0001) (Figure 1A). Cell viability was significantly reduced by allulose (~9%; *p* = 0.0001), glucose (~23%; *p* < 0.0001), and fructose (~58%; *p* < 0.0001) compared with controls (Figure 1B).

**Figure 2:**
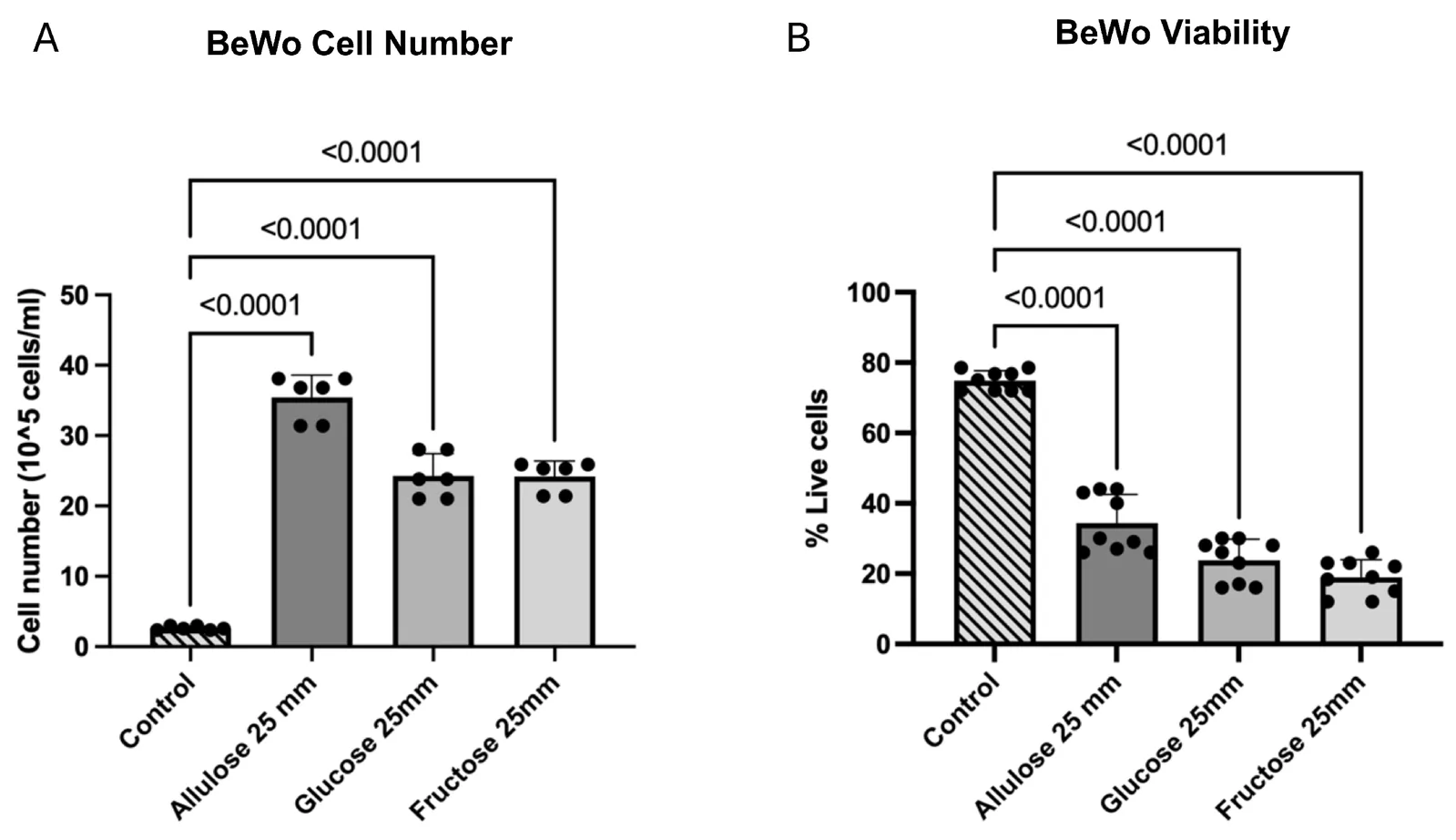
Effects of glucose, allulose, and fructose on BeWo trophoblast cell number and viability. BeWo cells were exposed for 24 h to glucose, allulose, or fructose added at 25 mM to standard medium (DMEM/F12, 17.5 mM basal glucose); controls received standard medium alone. (**A**) All sugar treatments significantly increased total cell number compared with control. (**B**) Cell viability was significantly reduced by all three sugars. Individual data points are shown as black dots. Data are mean ± SEM (n = 10). Statistical significance was determined by Kruskal–Wallis test with Dunn’s post hoc correction.

In BeWo cells, all treatments also increased cell counts compared with controls, with the largest increase following allulose (~15-fold; *p* < 0.0001) compared with glucose and fructose (~10-fold each; *p* < 0.0001) (Figure 2A). Because changes in this magnitude in either cell line exceed what cell division can produce within 24 h, we interpret them as changes in the number of cells recovered at harvest, not as proliferation. They may reflect treatment effects on adhesion, detachment, or aggregation, which the present data cannot separate. In contrast, cell viability decreased with all treatments compared with controls (~55% with allulose, ~69% with glucose, and ~75% with fructose; *p* < 0.0001) (Figure 2B).

### 2.2. Sugar Treatments Induce Oxidative Stress in Trophoblast Cells

**Figure 3:**
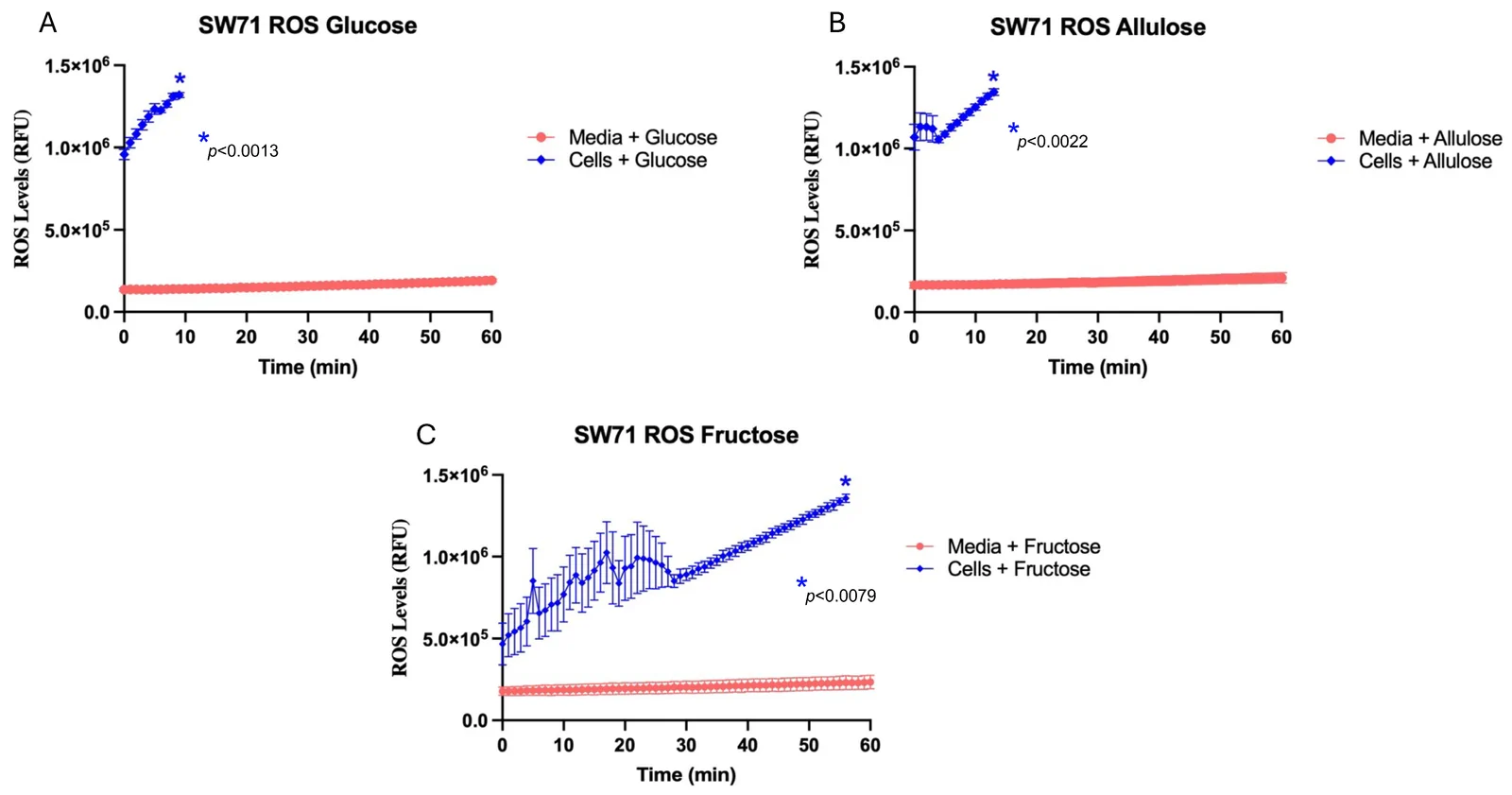
Sugar-induced oxidative stress in SW71 trophoblast cells. Intracellular ROS levels were measured in SW71 cells after 24 h treatment with (**A**) glucose, (**B**) allulose, or (**C**) fructose added at 25 mM to standard medium using the DCFH-DA assay. Red lines represent media containing the respective sugar without cells; blue lines represent cells incubated with the sugar. Blue traces end at the last reading within the detector’s linear range; later readings exceeded the upper detection limit. All sugars significantly increased ROS production compared with media-only controls. Data are mean ± SEM relative fluorescence units (RFU; n = 9). Statistical comparisons were made using the Mann–Whitney U test. * *p* < 0.05 vs. untreated control at the indicated time points.

**Figure 4:**
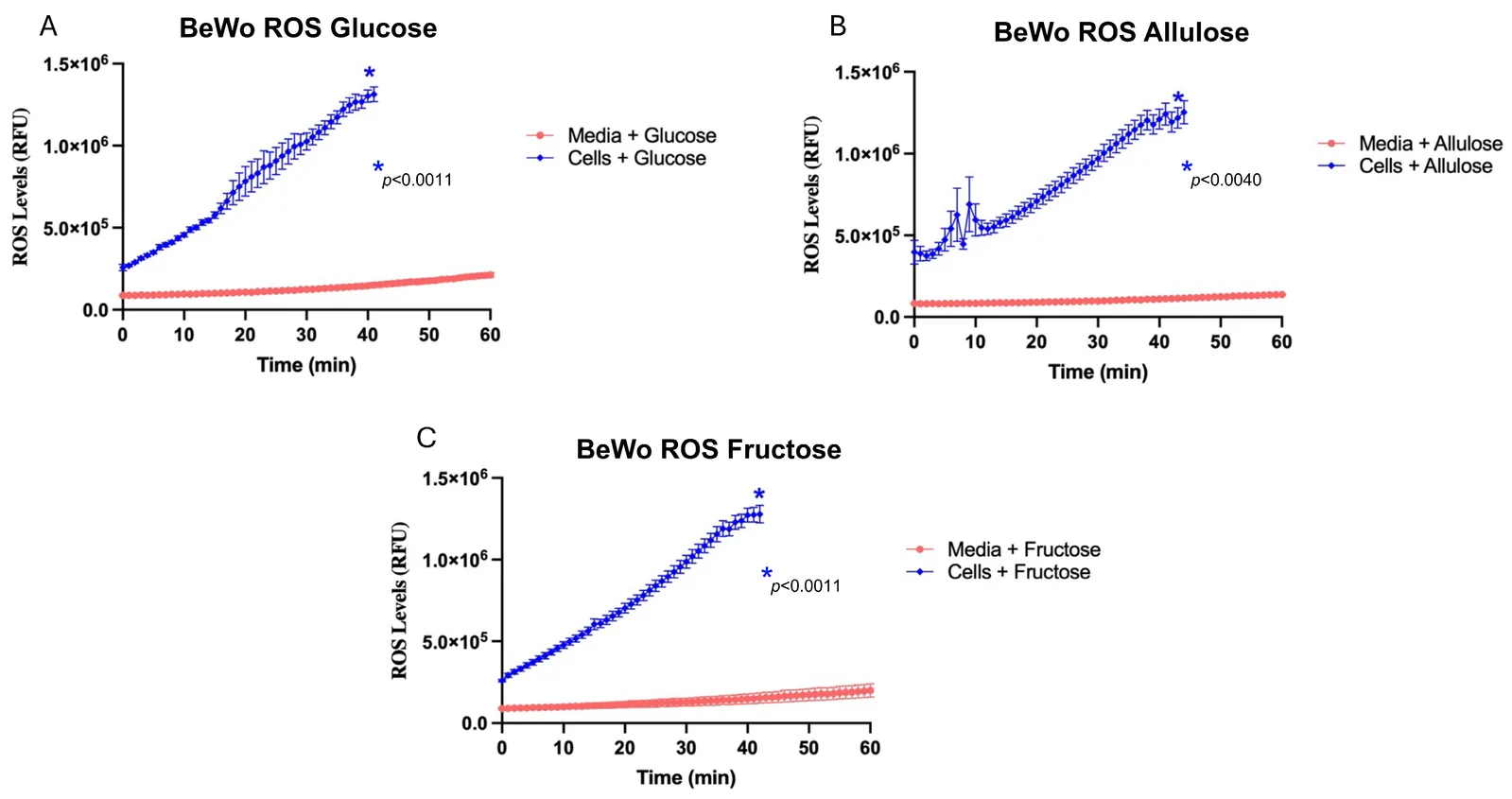
Sugar-induced oxidative stress in BeWo trophoblast cells. BeWo cells were treated for 24 h with (**A**) glucose, (**B**) allulose, or (**C**) fructose added at 25 mM to standard medium, and intracellular ROS levels were measured using DCFH-DA. Red lines show media + sugar (no cells); blue lines show cells + sugar. Blue traces end at the last reading within the detector’s linear range; later readings exceeded the upper detection limit. Each sugar significantly elevated ROS compared with controls. Data are mean ± SEM RFU (n = 9). Statistical significance was assessed by Mann–Whitney U test. * *p* < 0.05 vs. untreated control.

To assess cellular oxidative stress responses, we measured intracellular reactive oxygen species generation in SW71 and BeWo trophoblast cells following exposure to different sugars. Fluorescence intensity was monitored as a readout of ROS accumulation. Because DCF fluorescence accumulates over time, signal in several cell-containing wells exceeded the linear range of the plate reader before 60 min; these readings were excluded, so the blue traces in Figure 3 and Figure 4 end at different times (approximately 10–15 min for glucose- and allulose-treated SW71 cells, ~56 min for fructose-treated SW71 cells, and ~40–45 min for BeWo cells).

Values are means of all in-range readings (Section 4.3). In SW71 cells, treatment with glucose, allulose, and fructose all induced substantial ROS production compared to media-only controls. Control cells maintained baseline fluorescence values averaging 1.85 × 10^5^ ± 1.8 × 10^3^ RFU (mean ± SEM). Glucose treatment elevated ROS to 1.18 × 10^6^ ± 3.83 × 10^4^ RFU, representing a 6.3-fold increase over baseline (*p* = 0.0013; Figure 3A). Allulose exposure similarly increased ROS levels to 1.18 × 10^6^ ± 2.50 × 10^4^ RFU, a 6.4-fold elevation (*p* = 0.0022; Figure 3B). Fructose treatment resulted in ROS levels of 9.62 × 10^5^ ± 2.83 × 10^4^ RFU corresponding to a 5.2-fold increase above controls (*p* = 0.0079; Figure 3C).

BeWo cells showed similar patterns of oxidative stress. Media-treated controls exhibited baseline ROS values of 1.03 × 10^5^ ± 2.23 × 10^3^ RFU (mean ± SEM). Glucose exposure increased ROS to 7.73 × 10^5^ ± 5.20 × 10^4^ RFU, a 7.5-fold rise from baseline (*p* = 0.0011; Figure 4A). Allulose treatment produced the highest response at 8.02 × 10^5^ ± 4.21 × 10^4^ RFU, representing a 7.8-fold induction (*p* = 0.0040; Figure 4B). Fructose similarly elevated ROS to 7.61 × 10^5^ ± 4.88 × 10^4^ RFU, a 7.4-fold increase over controls (*p* = 0.0011; Figure 4C).

### 2.3. Sugar Treatments Differentially Modulate Mitochondrial Respiration in Trophoblast Cells

To evaluate mitochondrial bioenergetics, we assessed oxygen consumption rates in SW71 and BeWo trophoblast cells under sequential substrate additions. Cells were exposed to glutamate and malate to measure leak respiration, followed by ADP addition to stimulate phosphorylation-driven respiration, and finally succinate to engage both Complex I and Complex II pathways.

**Figure 5:**
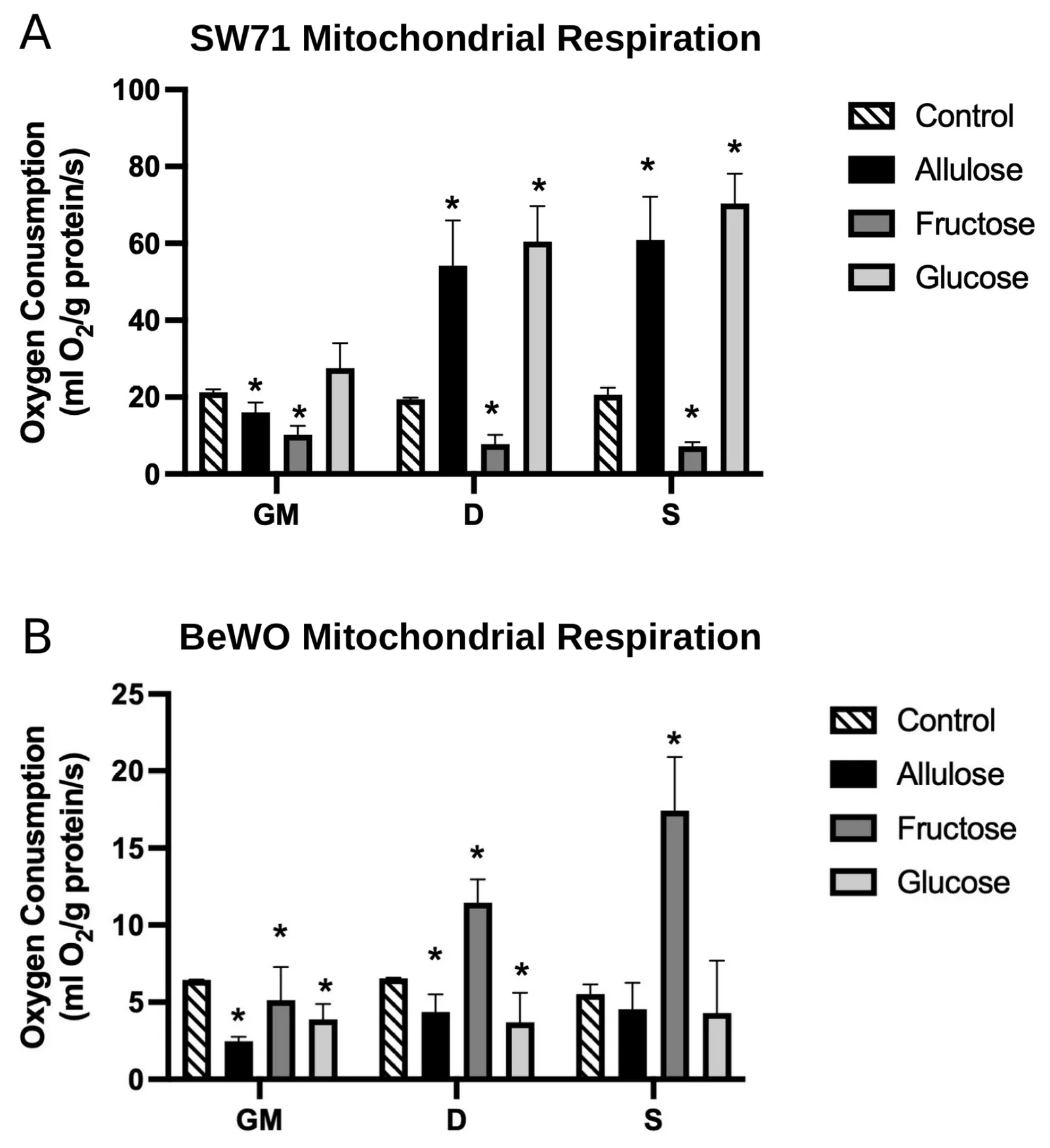
Differential effects of sugars on mitochondrial respiration in trophoblast cells. Oxygen consumption rates were measured in (**A**) SW71 and (**B**) BeWo cells after 24 h exposure to glucose, allulose, or fructose added at 25 mM to standard medium using high-resolution respirometry. GM = glutamate + malate (leak respiration); D = ADP-stimulated respiration; S = succinate-supported respiration (Complex I + II). Respiration rates were normalized to cell number. Individual data points are shown. Data are mean ± SEM (n = 6). Statistical analysis was performed using the Kruskal–Wallis test with Dunn’s post hoc correction. * *p* < 0.05 vs. control.

In SW71 cells, sugar treatments produced distinct effects on mitochondrial oxygen consumption across all respiratory states. During leak respiration (glutamate/malate), allulose and fructose significantly reduced oxygen consumption compared with controls (*p* < 0.05), whereas glucose did not differ from control (Figure 5A). Upon ADP addition, glucose and allulose increased respiration approximately three-fold relative to controls (*p* < 0.05), whereas fructose reduced it (*p* < 0.05). The same pattern was observed after succinate addition to engage Complex II (*p* < 0.05; Figure 5A). Thus, in the ADP-stimulated and succinate-supported states, fructose acted in the opposite direction to glucose and allulose.

In BeWo cells, the sugars produced a different pattern. During leak respiration, all three sugars reduced oxygen consumption relative to control (*p* < 0.05; Figure 5B). With ADP-stimulated phosphorylation, fructose markedly increased respiration compared with controls (*p* < 0.05), whereas allulose and glucose reduced it (*p* < 0.05; Figure 5B). Following succinate addition, fructose treatment sustained a pronounced elevation in oxygen consumption (*p* < 0.05), while allulose and glucose responses remained comparable to control (Figure 5B). Fructose therefore had opposite effects on ADP-stimulated and succinate-supported respiration in the two cell lines, suppressing it in SW71 cells and increasing it in BeWo cells.

### 2.4. Differential Effects of Sugar Treatments on Trophoblast Invasion

To determine whether different sugars exert distinct effects on trophoblast invasive capacity, we performed Matrigel invasion assays on SW71 extravillous trophoblast cells exposed to glucose, allulose, or fructose. This approach allowed direct comparison of how various dietary sugars modulate the fundamental invasive properties critical for placental development.

**Figure 6:**
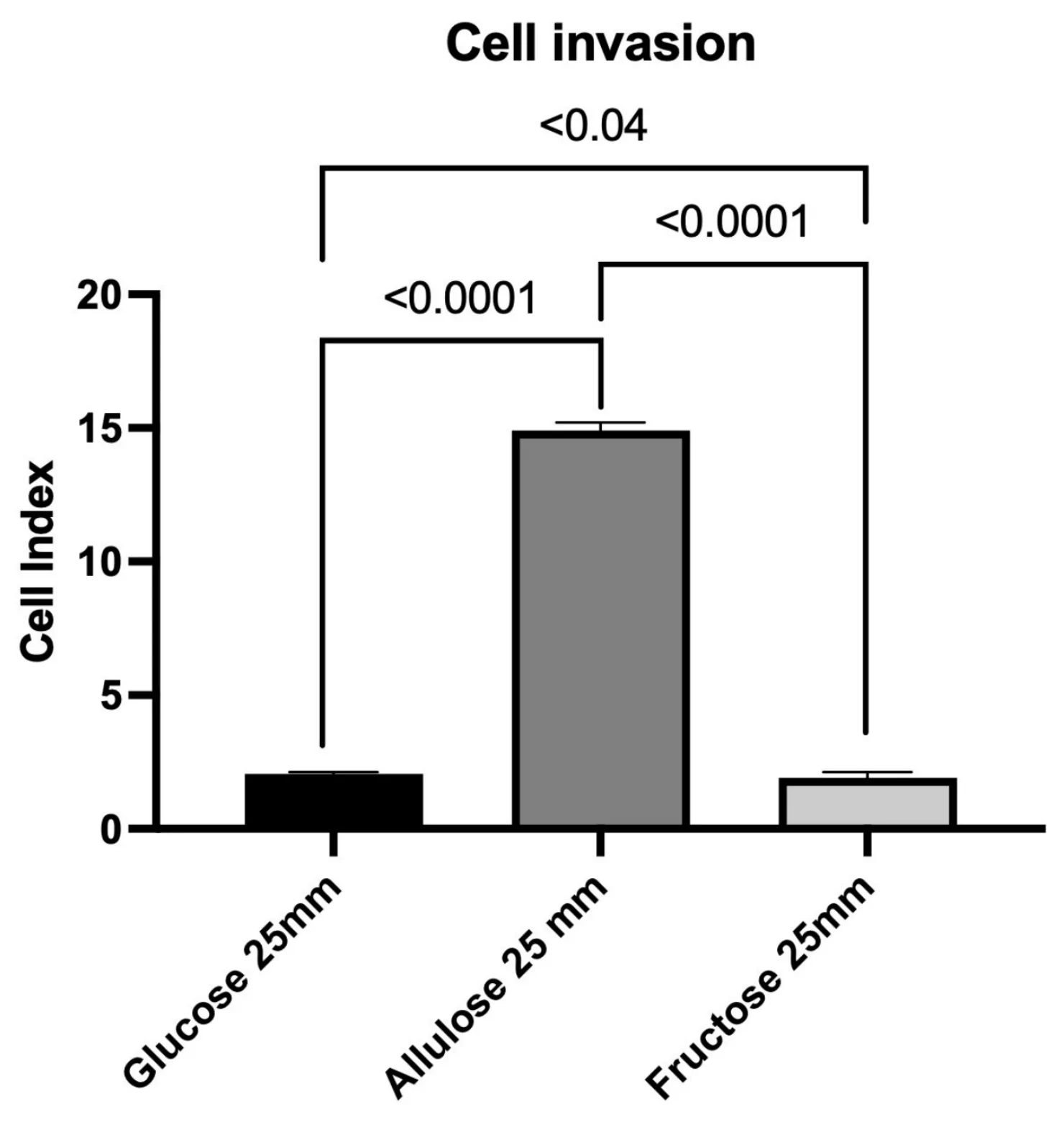
Sugar-specific modulation of trophoblast invasion. Real-time Matrigel invasion of SW71 extravillous trophoblast cells was assessed after 24 h treatment with glucose, allulose, or fructose added at 25 mM to standard medium. Invasion was greater after allulose than after glucose or fructose treatment. Individual data points are shown as black dots. Data are mean ± SEM invasion index (n = 6). Statistical significance was determined by Kruskal–Wallis test with Dunn’s post hoc correction. *p* < 0.05 vs. allulose.

Invasion was highest after allulose treatment. Invasion indices after glucose and fructose were ~7-fold lower than after allulose (*p* < 0.0001; Figure 6) and were slightly lower after fructose than after glucose (*p* < 0.04). Because an untreated control was not included in this assay, these data show differences between the sugars and do not establish whether allulose maintained invasion at control levels.

## 3. Discussion

We compared glucose, fructose, and allulose in two transformed trophoblast cell lines, which approximate but do not reproduce normal trophoblast physiology, under a fixed, supraphysiological sugar load. In both lines, adding 25 mM of each sugar to standard medium for 24 h increased the number of cells recovered at harvest and reduced viability. In SW71 cells, the increase was greatest with glucose (6.5-fold), followed by allulose (3.2-fold) and fructose (2.4-fold); in BeWo cells, it was greatest with allulose (~15-fold) and similar for glucose and fructose (~10-fold). Viability fell by ~9% with allulose, ~23% with glucose, and ~58% with fructose in SW71 cells and by ~55–75% in BeWo cells, with fructose producing the largest loss in both lines. Because these changes exceed what cell division can achieve in 24 h and were measured only by counting and Trypan Blue exclusion, they cannot be attributed to proliferation or to a specific form of cell death; proliferation and apoptosis markers will be needed to resolve them. The greater loss of viability with fructose is consistent with, but was not shown to result from, ATP depletion and uric acid production during its metabolism by fructokinase [19].

All three sugars increased DCF fluorescence in both lines (5.2- to 7.8-fold), with little difference between sugars. Oxidative stress is a recognized feature of placentas from pregnancies complicated by GDM [20,21], and high fructose intake induces placental oxidative stress in rodents [22,23]. DCFH-DA reports general oxidant activity rather than a specific species or source, and values were not normalized to cell number or protein, so these data show that the sugars raised oxidant load but do not identify its source; mitochondrial electron leak and NADPH oxidase activity are possible sources that we did not test. Allulose raised ROS as much as glucose, indicating that at this concentration it did not protect trophoblast cells against oxidative stress.

Oxygen consumption differed between sugars within each line. In SW71 cells, glucose and allulose increased ADP-stimulated and succinate-supported oxygen consumption about three-fold, whereas fructose reduced oxygen consumption in all states measured. In BeWo cells, fructose was the only sugar that increased oxygen consumption in these states. The opposite effect of fructose in SW71 cells is consistent with evidence that human cells handle fructose poorly: it is phosphorylated by fructokinase and enters glycolysis downstream of the phosphofructokinase control step, and high fructose intake has been linked to metabolic syndrome, inflammation, and adverse pregnancy and offspring outcomes [19,24,25]. The increase with allulose, which is minimally metabolized, suggests that this change in oxygen consumption is not driven by substrate supply. Because rates were normalized to cell number rather than mitochondrial content, were not corrected for residual oxygen consumption, and did not allow calculation of coupling parameters, these data describe changes in oxygen consumption only; they do not establish mitochondrial dysfunction in SW71 cells or compensatory adaptation in BeWo cells. Whether the opposite effect of fructose in the two lines reflects lineage, transformation status, or basal glucose cannot be resolved with the present design.

In SW71 cells, invasion was ~7-fold higher after allulose than after glucose or fructose. High glucose reduces trophoblast invasion in vitro [26], and glucose and fructose might act through integrins, matrix metalloproteinases, or, for fructose, uric acid [24]; none of these was measured. The higher invasion after allulose occurred despite a comparable rise in ROS and a loss of viability, so it is not explained by lower oxidative stress. How trophoblasts respond to a minimally metabolized sugar is unknown. Candidate routes, which we offer only as hypotheses, include transport by GLUT11, whose placenta-enriched isoform is expressed in human placenta [27], and signaling through sweet-taste receptors (T1R2/T1R3), which sense sugars in some non-gustatory tissues [28] but have not been described in human trophoblasts; testing them will require expression profiling, inhibition, and knockdown studies. Because the assay lacked an untreated control, we cannot say whether allulose maintained invasion at baseline levels.

Syncytialization is central to placental endocrine and transport function, and its disruption is a plausible link between maternal diabetes and pregnancy disease. In forskolin-stimulated BeWo cells, 25 mM glucose reduced β-hCG and syncytin-2 expression and the fusion index compared with 5.5 mM glucose, and placentas from pregnancies complicated by type 2 diabetes showed impaired syncytialization [29]. Similarly, a metabolic-stress cocktail of high glucose, insulin, and palmitate suppressed syncytin expression and β-hCG and progesterone secretion in syncytialized BeWo cells [30]. Whether fructose or allulose affects fusion is unknown. Because our BeWo experiments used undifferentiated, cytotrophoblast-like cells, our findings do not address the syncytiotrophoblast, and this is the most important question arising from this study. Future work should test forskolin-induced fusion under each sugar, measuring β-hCG secretion, the fusion index, and syncytin-1 and syncytin-2 expression, in BeWo cells and primary cytotrophoblasts.

These findings should be related to pregnancy with caution. Total glucose in the glucose-treated wells was approximately 36–42.5 mM, about four times the concentrations seen even in poorly controlled GDM (~8–10 mM), and circulating fructose and allulose concentrations after dietary intake are far below 25 mM. The design therefore compares sugars under a fixed, supraphysiological stress and does not model maternal glycemia or dietary sugar intake. This caution applies particularly to allulose. Although allulose-treated SW71 cells invaded more than glucose- or fructose-treated cells, allulose also increased ROS, reduced viability in both lines, and produced the largest increase in BeWo cell recovery, and only one concentration was tested. These data therefore do not indicate that allulose is safer than other sugars for placental function and should not inform dietary advice in pregnancy without dose–response studies at physiological concentrations and in vivo data. By comparison, the effects of fructose on viability, oxygen consumption, and invasion agree with animal studies linking high maternal fructose intake to placental oxidative stress and adverse offspring metabolism [22,25,31].

This study has several limitations. First, BeWo and SW71 are transformed cell lines with substantial genomic alterations, cultured at atmospheric oxygen (~18–20%); undifferentiated BeWo cells are cytotrophoblast-like, and neither line fully reproduces primary trophoblasts. The findings are therefore comparative and hypothesis-generating and require validation in primary trophoblasts or trophoblast organoids. Second, sugars were added to standard media that already contained 11–17.5 mM glucose, so controls were not normoglycemic; the response to added glucose may be blunted because GLUT1-mediated uptake, which has a low-millimolar affinity for glucose, is likely near saturation, and the two lines differed in basal glucose, which limits comparisons between them. Third, a single supraphysiological concentration was tested and no iso-osmotic mannitol control was included, so differences between each sugar and the control cannot be separated from the ~8% increase in osmolality, although differences between sugars can. Fourth, cell number and viability were assessed only by counting and Trypan Blue exclusion, without proliferation or cell-death markers; cell number was not verified at the start of treatment, and no images of the cultures were acquired. Fifth, respirometry lacked a residual-oxygen step and normalization to mitochondrial content, and ROS values were not normalized to protein or viable cell number. Sixth, we did not examine whether the sugars alter BeWo fusion or syncytiotrophoblast endocrine function, such as forskolin-induced hCG secretion; because undifferentiated BeWo cells are cytotrophoblast-like, our BeWo data do not address the syncytiotrophoblast. Future work will test sugar effects on forskolin-induced fusion and will use glucose-free media reconstituted with 5 mM glucose and a mannitol control, a physiological dose range, primary trophoblasts or organoids, proliferation and apoptosis assays, residual-oxygen-corrected respirometry normalized to citrate synthase activity, and profiling of candidate sugar transporters and receptors.

## 4. Materials and Methods

### 4.1. Cell Culture and Experimental Treatments

Human trophoblast cell models representing distinct placental phenotypes were utilized, including BeWo cells (choriocarcinoma-derived, villous; ATTCC, Manassas, VA, USA) and SW71 cells (invasive cytotrophoblast-like). The SW71 cell line was generously provided by Dr. Gil Mor (Yale University). BeWo cells were maintained in DMEM/F12 (basal glucose ~17.5 mM; Fisher Scientific, Waltham, MA, USA), whereas SW71 cells were cultured in RPMI medium (basal glucose ~11 mM; Fisher Scientific, Waltham, MA, USA); both media were supplemented with 10% fetal bovine serum and 1% penicillin–streptomycin. Cells were incubated at 37 °C in a humidified atmosphere containing 5% CO_2_ under atmospheric oxygen (~18–20% O_2_). BeWo cells originate from choriocarcinoma and exhibit a strongly glycolytic phenotype. Under standard conditions they are largely mononuclear and cytotrophoblast-like; they fuse and acquire syncytiotrophoblast endocrine function, including hCG secretion, only when stimulated with forskolin. Syncytialization was not examined in this study, so BeWo cells here model undifferentiated villous trophoblast. SW71 cells are immortalized first-trimester extravillous trophoblasts that retain a more oxidative metabolism and retain invasive capacity. Because each line was maintained in its standard medium, the two lines differed in basal glucose (17.5 vs. 11 mM) as well as in origin and metabolic phenotype; we therefore compare sugar effects within each cell line and do not attribute differences between the lines to lineage alone. For each experiment, all wells were seeded from a single cell suspension at 2 × 10^6^ cells per well. Upon reaching approximately 80% confluence, cells were exposed for 24 h (n = 10) to glucose, allulose, or fructose added at 25 mM to their standard medium. Control cells were maintained in the same standard medium without added sugar. Throughout the manuscript, “25 mM” denotes the added concentration; all conditions, including controls, therefore contained the basal glucose of the respective medium. Cell viability was assessed using Trypan Blue exclusion and quantified as the percentage of viable cells relative to total cell count. Total and viable cell numbers were determined using an automated cell counter with Trypan Blue exclusion, with total cell number recorded as the sum of viable and non-viable cells. The 25 mM concentration was selected to model conditions of metabolic stress rather than physiological normoglycemia and is consistent with concentrations commonly used to study trophoblast responses under diabetic-like conditions in vitro. We acknowledge that this concentration exceeds typical maternal glucose levels in vivo and may incorporate osmotic stress; however, all treatment groups were exposed to equivalent molar concentrations of sugar to allow direct comparison of sugar-specific cellular responses. Accordingly, the conclusions of this study are limited to comparative, sugar-specific responses under matched hyperosmolar stress conditions rather than absolute metabolic effects. This concentration exceeds glucose levels observed even in poorly controlled gestational diabetes (~8–10 mM) and was therefore not intended to replicate maternal glycemia [26,32]. The added monosaccharide raised medium osmolality by approximately 25 mOsm/kg (~8% above basal medium), a modest hyperosmotic increment applied identically to all treatment groups; the treatment groups are thus equiosmolar to one another, and osmotic load cannot account for the differences observed between sugars. Because no iso-osmotic (mannitol) control was included, however, differences between each sugar and the untreated control cannot be separated from this increase in osmolality. Because the basal media themselves contained glucose (~17.5 mM in DMEM/F12; ~11 mM in RPMI), the added-glucose condition represents an incremental rise above a glucose-replete background, whereas the fructose and allulose conditions add a distinct monosaccharide to that same background; fructose- and allulose-specific effects are therefore interpreted relative to this shared glucose-containing baseline. Taking the basal media into account, total glucose in the supplemental-glucose condition reached approximately 42.5 mM (BeWo) and 36 mM (SW71), whereas the fructose and allulose conditions added 25 mM of a distinct monosaccharide to the same glucose-containing background.

### 4.2. Assessment of Mitochondrial Respiration

Mitochondrial function was evaluated (n = 6) by measuring oxygen consumption rates in treated and untreated trophoblast cells using a high-resolution Oroboros O2k Oxygraph (Oroboros Instruments, Innsbruck, Austria). Cells were collected after treatment and resuspended in MiR05 respiration buffer, and measurements were conducted at 37 °C. Cells were permeabilized before substrate addition so that substrates and ADP had direct access to the mitochondria.

Respiratory parameters were determined using a substrate–uncoupler–inhibitor titration protocol. Respiration was first measured after addition of glutamate (10 mM) and malate (2 mM) in the absence of ADP, corresponding to the NADH-linked LEAK state (GM in Figure 5), in which oxygen consumption is driven mainly by proton leak rather than ATP synthesis. Adenosine diphosphate (2.5 mM) was subsequently introduced to stimulate oxidative phosphorylation. Succinate (10 mM) was then added to enable convergent electron input through complexes I and II. Maximal electron transport system capacity was assessed following stepwise titration of the uncoupler carbonyl cyanide 4-(trifluoromethoxy) phenylhydrazone (FCCP; titrated to achieve maximal uncoupling). This approach allowed descriptive comparison of oxygen consumption across respiratory states between treatment groups and controls. Respiration rates were normalized to cell number, and experiments were performed in duplicate or triplicate across at least three independent runs. Respiration rates were normalized to cell number rather than to a marker of mitochondrial content such as citrate synthase activity or mtDNA copy number. The protocol did not include inhibition with rotenone and antimycin A, so rates were not corrected for residual (non-mitochondrial) oxygen consumption, and the respiratory control ratio and coupling efficiency were not calculated. Oxygen consumption is therefore compared descriptively between treatments within each cell line.

### 4.3. Measurement of Intracellular Reactive Oxygen Species

Intracellular reactive oxygen species (ROS) generation was quantified using the redox-sensitive fluorescent dye 2′,7′-dichlorodihydrofluorescein diacetate (DCFH-DA; Sigma-Aldrich, St Louis, MO, USA), following the manufacturer’s recommendations with minor modifications. SW71 and BeWo trophoblast cells were seeded at a density of 1 × 10^4^ cells per well in black, clear-bottom 96-well plates (Corning, Tewksbury, MA, USA, Cat. No. 3603) and allowed to adhere overnight in their respective complete growth media (SW71: RPMI with 10% fetal bovine serum; BeWo: DMEM/F12 with 10% fetal bovine serum). SW71 and BeWo cells were treated with glucose, allulose, or fructose added at 25 mM to standard medium, or with standard medium alone, for 24 h. A 10 µM DCFH-DA working solution was freshly prepared in Hank’s Balanced Salt Solution (HBSS) from a 10 mM stock solution in DMSO stored at −20 °C in light-protected aliquots. At the end of the 24 h sugar treatment, cells were loaded with 100 µL of the freshly prepared 10 µM DCFH-DA working solution per well for 45 min at 37 °C in the dark to allow intracellular probe loading. Wells were then gently washed twice with PBS to remove residual dye, and fluorescence was recorded at 37 °C every 1 min for up to 60 min (excitation 485 nm; emission 535 nm) using a BioTek Synergy H1 microplate reader (Biotek; Winooski, VT, USA). Readings that exceeded the upper limit of the detector’s linear range (reported by the instrument as overflow; ~1.3 × 10^6^ RFU) were excluded, so each kinetic trace ends at its last in-range reading. ROS values reported in the text are the mean of all in-range readings for each condition; because the in-range window differed between conditions, these values summarize overall fluorescence rather than a rate at a single time point. Hydrogen peroxide (100 µM), added at the time of treatment, served as a positive control. Additional control wells containing no cells and either glucose, allulose, or Fructose alone were included to assess background signal. Fluorescence values were reported as relative fluorescence units (RFU) following subtraction of blank readings obtained from HBSS-only wells. RFU were not normalized to cell number or protein. Each condition was analyzed in triplicate wells across three independent experiments for a total of n = 9 replicates. We note that this probe provides a general readout of oxidative stress and can interact with several reactive species rather than one specific type.

### 4.4. Real-Time Assessment of Trophoblast Invasion

The invasive behavior of trophoblast cells following exposure to glucose, allulose, or fructose (n = 6 per condition) was assessed using a real-time impedance-based invasion assay, as previously established in our laboratory. Cell invasion was monitored using the xCELLigence RTCA DP system (ACEA Biosciences; San Diego, CA, USA) and CIM-16 plates. Prior to cell seeding, the upper chambers were coated with Matrigel diluted 1:40 in serum-free medium to mimic extracellular matrix conditions. A total of 20,000 cells suspended in RPMI containing 2% fetal bovine serum were added to the upper chamber, while the lower chamber contained complete medium supplemented with 10% fetal bovine serum to generate a chemoattractant gradient. Electrical impedance, reflecting cell movement through the Matrigel layer, was recorded every 15 min for 24 h. Invasion indices were calculated using the RTCA software based on impedance changes over time. Invasion assays were performed exclusively with SW71 cells, which model the invasive extravillous trophoblast population responsible for spiral artery remodeling in vivo. BeWo cells largely lack this invasive behavior in Matrigel-based assays and were therefore not included in these experiments.

### 4.5. Statistical Analysis

Data are presented as mean ± standard error of the mean (SEM). The normality of the data distribution was assessed prior to statistical testing. Although normality was evaluated, given the relatively small sample sizes typical of cell culture experiments (n = 6–10 per group) and the inherent biological variability across independent cell passages and experimental runs, non-parametric tests (Mann–Whitney U test for pairwise comparisons or Kruskal–Wallis test with Dunn’s post hoc for multiple groups) were selected as a conservative approach. A *p*-value of less than 0.05 was considered statistically significant. All statistical analyses were conducted using GraphPad Prism version 8.0 (GraphPad Software, San Diego, CA, USA). Throughout, n refers to the number of independent biological replicates (experiments performed on separate occasions); technical replicate wells within each experiment were averaged prior to analysis.

## 5. Conclusions

Under the in vitro conditions used here, glucose, fructose, and allulose were not interchangeable in their effects on trophoblast cells. All three increased cell recovery and ROS, but they differed in their effects on viability, oxygen consumption, and SW71 invasion. Fructose caused the largest loss of viability and reduced oxygen consumption and invasion in SW71 cells, whereas allulose-treated SW71 cells invaded more than glucose- or fructose-treated cells. Because BeWo cells were studied without induced fusion, these results do not address the syncytiotrophoblast, and testing sugar effects on syncytialization is a priority. These results are hypothesis-generating and support testing sugar-specific effects on the placenta at physiological concentrations and in primary trophoblast models.

## Data Availability

The original contributions presented in the study are included in the article.

## Abbreviations

The following abbreviations are used in this manuscript:

ADP	adenosine diphosphate.
CTB	cytotrophoblast.
DCFH-DA	2′,7′-dichlorodihydrofluorescein diacetate.
DMSO	dimethyl sulfoxide EVT: extravillous trophoblast.
FCCP	carbonyl cyanide 4-(trifluoromethoxy)phenylhydrazone.
GDM	gestational diabetes mellitus.
GLUT	glucose transporter.
HBSS	Hank’s Balanced Salt Solution.
RFU	relative fluorescence units.
ROS	reactive oxygen species.
RTCA	real-time cell analysis.
SEM	standard error of the mean.
STB	syncytiotrophoblast SW71: Swan71 cells.
